# User-centered Design of an Adjunct Smartphone App to Reduce Cannabis Use among Youth Diverted from the Juvenile Legal System

**DOI:** 10.1007/s10802-025-01370-6

**Published:** 2025-09-09

**Authors:** Sarah A. Helseth, Kaitlin N. Piper, Christopher J. Dunne, Kathleen Kemp, Nancy P. Barnett, Melissa A. Clark, Anthony Spirito, Sara J. Becker

**Affiliations:** 1 Center for Dissemination and Implementation Science, Northwestern University Feinberg School of Medicine, Chicago, IL USA; 2 Department of Psychiatry and Behavioral Sciences, Northwestern University Feinberg School of Medicine, Chicago, IL USA; 3 Department of Behavioral, Social, and Health Education Sciences, Emory University Rollins School of Public Health, Atlanta, GA USA; 4 Department of Psychiatry and Human Behavior, Alpert Medical School, Brown University, Providence, RI USA; 5 Rhode Island Hospital, Providence, RI USA; 6 Department of Behavioral and Social Sciences, School of Public Health, Brown University, Providence, RI USA; 7 Department of Health Services Policy & Practice, School of Public Health, Brown University, Providence, RI USA

**Keywords:** Diverted youth, Juvenile legal system, Digital health, Cannabis, Intervention development

## Abstract

Cannabis use is common among US youth who become involved in the juvenile legal system (JLS), yet substance use treatment rates remain low, particularly among youth diverted away from formal JLS involvement. Diverted youth encounter multiple barriers to receiving services in the community that could be addressed via digital approaches offered by the JLS. This multiphase work details development of the TECH (Teen Empowerment through Computerized Health) app, a tailored digital adjunct to usual JLS services. First, qualitative interviews with diverted youth (*n* = 14) aged 14–18 years and their caregivers (*n* = 8) established youths’ cannabis-related treatment needs and preferences; youth were asked to specifically consider several theory-driven app components (personalization, behavior change, social interaction, gamification, and motivation enhancement). After building the TECH app, 10 diverted youth beta-tested the prototype over one month, to inform its preliminary feasibility (i.e., participation and app metadata) and acceptability (i.e., app quality and user satisfaction). Qualitative results indicated diverted youth and caregivers were open to an app to reduce cannabis use. Interviewed youth were divided about theoretically driven features, likely due to their unique preferences and needs. Prototype beta testing demonstrated diverted youth were willing to engage; metadata indicated all beta-testers used TECH independently after their baseline appointment, demonstrating feasibility. Participants rated satisfaction as slightly above average, reporting the app was of good quality and recommending ways to increase acceptability. We discuss implications of these preliminary findings on the app itself and the broader field of digital health for youth diverted from the JLS.

**Supplementary Information:**

The online version contains supplementary material available at 10.1007/s10802-025-01370-6.

While adolescents in the United States (US) most often use alcohol or nicotine (Substance Abuse and Mental Health Services Administration, 2024), cannabis is the most commonly used substance among youth involved in the juvenile legal system (JLS; Dir et al., 2020). Many negative long-term outcomes can be linked to early-onset cannabis use, including the development of substance use (SU) disorders (Winters & Lee, 2008), later criminal legal system involvement (Fergusson et al., 2002; Fergusson et al., 2002), and violent behavior (Brady et al., 2008). Cannabis use is a significant risk factor for re-arrest and detention among JLS-involved youth (Tolou-Shams et al., 2014). Together, the synergistic, maladaptive influences of cannabis use and JLS involvement (Adelman, 2020; Chassin et al., 2009) represent a public health concern in need of targeted early interventions.

Critically, more than 80% of youth who engage with the JLS are diverted away from deeper system involvement (Puzzachera et al., 2022). Juvenile diversion programs allow some youth to avoid formal court processes, like being charged with an offense, adjudicated, or detained, to minimize harmful effects of JLS involvement (Farn, 2018). Diverted youth typically remain in their communities and can receive referrals for a wide range of services, including SU treatments (Wasserman et al., 2021). Families of diverted youth often face a myriad of competing demands that can diminish engagement with community-based treatments (Bath et al., 2018; Johnson-Kwochka et al., 2022), even when services are mandated by the court (i.e., must be completed to avoid formal escalation of their case) or offered in the home, increasing their risk of facing further legal consequences as well as SU problems. Recent evidence indicates fewer than 30% of diverted youth actually receive the SU services they need (Dennis et al., 2019), suggesting systems-level implementation barriers. For example, most JLS agencies were not designed to deliver secondary prevention services and lack a workforce trained to deliver SU treatments. Furthermore, numerous studies have documented systematic racial and ethnic disparities in referrals to (Spinney et al., 2016) and completion of SU services among diverted youth in the US (Marotta et al., 2022). Given these systemic inequities and system-level challenges, any new efforts to reduce cannabis use among JLS-involved youth would, by design, need to maximize equity and accessibility, minimize resource demands, and be effective.

Intensive, home-based interventions like multisystemic therapy (Henggeler & Schaeffer, 2019) and multidimensional family therapy (Liddle, 2016) are effective at reducing SU and other justice-related outcomes (e.g., recidivism; for review, see Dauria et al., 2018). However, intensive approaches require a substantial time commitment from families of diverted youth and possibly a financial investment from the JLS, such that they are generally most appropriate for those youth with the most severe behavioral health needs. Alternatively, brief intervention approaches, such as motivation enhancement therapy + cognitive-behavioral therapy, have demonstrated small but significant treatment effects on cannabis use in adolescent samples that included JLS-involved youth (Dennis et al., 2004). Even so, brief SU interventions often require multiple sessions (Steele et al., 2020) and may not be appropriate for youth whose cannabis use has not reached the level of a disorder (e.g., developmentally appropriate experimentation; Sultan et al., 2023), who might benefit from less-intensive secondary prevention approaches.

As research on secondary prevention interventions to reduce cannabis use among diverted youth is in its infancy, looking to theory and evidence from the broader adolescent SU literature can offer some valuable insights. Theories of adolescent SU (Petraitis et al., 1995) largely center on key intrapersonal and interpersonal mechanisms of behavior. Intrapersonal mechanisms encompass one’s beliefs, attitudes, self-efficacy, and expectancies about SU (Brook et al., 1999; Brown et al., 1987; Ebersole et al., 2014; Feinstein et al., 2012; Handley & Chassin, 2009), while interpersonal mechanisms reflect external influences, like perceptions of peers’ SU (i.e., peer norms), social reinforcement or behavioral modeling (Dishion et al., 1996; Dishion & Andrews, 1995; Hansen & Graham, 1991; Petraitis et al., 1995). While most established SU interventions aim to mitigate rather than leverage interpersonal influences on SU, some have sought to activate protective interpersonal factors to achieve SU behavior change. Several brief computerized interventions have successfully reduced youth SU by correcting misperceptions of both peers’ SU and approval of SU behavior (Doumas et al., 2014). However, effects seem to fade over time (Doumas, Hausheer, Doumas et al., 2014a, b), suggesting a need for adjunctive supports to sustain clinical gains. Others have combined motivation-enhancement and accurate peer cannabis use norms, finding that participants reported more accurate perceived peer norms post-treatment (Blevins et al., 2018); changes in one’s own approval of cannabis use and in perceived peer norms were both positively correlated with post-treatment improvements in cannabis use and related outcomes. Though not specifically targeting JLS-involved youth, these findings highlight the potential utility of leveraging both intrapersonal and interpersonal mechanisms of SU behavior change in future interventions targeting youth cannabis use, perhaps even in the context of novel secondary prevention approaches for diverted youth.

Digital technologies offer a natural means of leveraging interpersonal mechanisms of behavior change in secondary prevention interventions, as most youth already engage with peers daily via technology (Pew Research Center, 2013). Digital approaches may also help address the systems-level barriers to SU treatment that diverted youth in JLS settings face, given their customizable, accessible, and portable format (Campbell et al., 2012; Grove et al., 2021; Linke et al., 2007; Prince et al., 2021; Riggs et al., 2018; Rooke et al., 2010). Mobile apps offer an ideal means of maintaining treatment gains, as they provide ongoing, on-demand adjunct to current or recent services diverted youth may receive. The ubiquity of smartphones lends further credence to mobile apps, as more than 91% of US youth have their own smartphone (Rideout & Robb, 2019) and national surveys report equitable smartphone access across adolescent racial, ethnic, gender, and income groups (Pew Research Center, 2024). As so-called digital natives (Prensky, 2001), youth use mobile apps more than adults and believe they are an acceptable, engaging, and confidential platform for health interventions (Black et al., 2014; Dennis et al., 2015).

Research to develop and test smartphone apps for adolescent SU that employ evidence-based practices or resources has grown dramatically in the past decade. Still, a 2021 systematic review of smartphone apps for SU (Kazemi et al., 2021) found just two adjunctive smartphone apps specifically for youth with cannabis use disorders. Both adjunctive apps employed ecological momentary assessment or intervention programming (Heron & Smyth, 2010) and demonstrated feasibility and efficacy (Dennis et al., 2015; Kells & Shrier, 2017; Shrier et al., 2018; Shrier, Rhoads, Burke, Shrier et al., 2014a, b; Shrier et al., 2014). However, both apps were designed for older adolescents receiving intensive, clinician-delivered treatments for heavy cannabis use, which does not reflect the bulk of untreated JLS-involved youth who use cannabis. Furthermore, neither of these apps for adolescent cannabis use, nor any other empirically tested apps for adolescent SU, have included any peer networking features or sought to leverage interpersonal mechanisms of adolescent SU behavior change, an important treatment consideration with adolescents (Van Ryzin et al., 2012) that has also been shown to be important with adults involved in drug court (Johnson et al., 2016). Despite the rapid growth in digital interventions for cannabis use since the current study’s launch, reflected in multiple published reviews reporting mixed evidence for reductions in cannabis use (Beneria et al., 2022; Côté et al., 2024; Sedrati et al., 2021), protocols for forthcoming trials (Coughlin et al., 2024; Tatar et al., 2022), and pilot trials reporting feasibility and acceptability (Santesteban-Echarri et al., 2021), no new research has focused on youth samples. Importantly, most existing apps, whether for SU or other behavioral health concerns, have not been tested in JLS-involved samples and no mobile app has ever been designed in partnership with diverted youth. Engaging youth in app development is essential (Lyon & Koerner, 2016; Mohr et al., 2017) to ensure that tailored digital interventions address not only the specific SU service needs of diverted youth but also the unique ethical and contextual constraints associated with JLS-delivered behavioral health services.

## The Current Study

Here, we describe three years and two phases of formative work to develop and beta-test the Teen Empowerment Through Computerized Health (TECH) app prototype. TECH was conceptualized as a digital adjunct to the typical services deployed by our partner family court (i.e., treatment-as-usual [TAU]) that could help diverted youth reduce their use of cannabis and other substances. To address gaps in the field, TECH is designed to: be highly accessible, focused on secondary prevention, and explicitly address both interpersonal and intrapersonal mechanisms of change. In Phase 1, we solicited feedback from diverted youth and their caregivers on our theory-driven anticipated app components to guide decision-making on both content and design of the app prototype. In Phase 2, 10 diverted youth beta-tested the TECH app prototype for one month. In addition to troubleshooting the prototype’s functionality, our goal was to assess the TECH app’s preliminary feasibility and acceptability prior to Phase 3’s pilot randomized trial; feasibility is commonly conceptualized as user engagement, either with the research (e.g., enrollment) or the app (e.g., app use metadata), whereas acceptability typically encompasses user satisfaction. Both Phase 1 and 2 were guided by principles of user-centered design (Lyon & Koerner, 2016; Mohr et al., 2017), which recommend engaging end users and other stakeholders in all aspects of design and refinement, to ensure the final product serves the needs and preferences of the target population.

## Methods

The published study protocol (Helseth et al., 2022) details all methods for this multiphase study. We first describe the setting, eligibility, and recruitment for Phases 1 and 2, before detailing phase-specific methods and results. All research activities were approved by the Brown University Institutional Review Board.

### Setting

The study was conducted in partnership with a statewide family court located in the Northeastern United States that annually serves an average of 2,400 youth facing a range of juvenile offenses. Adolescent participants and their caregivers were recruited exclusively from our partner’s court-supervised diversion program, which seeks to minimize youths’ contact with the JLS and avoid detention. The program is intended for youth with a first-time or less serious second status offense (offenses that can only be committed by a minor, e.g., truancy, underage drinking) or a first-time delinquent offense (criminal offenses committed by a minor, e.g., arson, breaking and entering, felony assault and/or battery). Following their intake assessment, family court staff assign restorative justice (Development Services Group INC, 2021) conditions that the diverted youth must meet in order for their charges to be dismissed. Family court staff also use evidenced-based screening practices for mental health and SU concerns, and can recommend or mandate (i.e., require as a part of the diversion process) a range of local behavioral health services options, including SU treatment, as well as opportunities to participate in clinical research. The decision of whether a youth was mandated to complete the court’s TAU for SU was at the sole discretion of the intake worker. Recent internal demographic data (reported in Helseth et al., 2024) indicate that the court’s juvenile population is predominantly 12–17 years old (98%) and identifies as male (71%) and Non-Hispanic White (62%), with over-representation of Black/African American (22%) youth relative to youth state-wide.

### Eligibility

To participate in Phase 1 or 2, diverted youth needed to: (1) be 14–18 years old, reflecting the stronger predictive value of peer influence on SU in late adolescence (Van Ryzin et al., 2012); (2) be able to speak and understand English; (3) have access to a smartphone; (4) report any past-year cannabis use during formal screening by the family court (i.e., the Massachusetts Youth Screening Instrument-Version 2 (Grisso & Barnum, 2006) or the “Car; Relax; Alone; Forget; Friend; Trouble” screener commonly referred to as the CRAFFT v 2.0 (Knight, 2016); and (5) be able to provide consent and/or assent. The research team confirmed eligibility during the initial recruitment call with youth. Caregivers were eligible for Phase 1 if they were: (1) the legal guardian of an eligible youth; and (2) able to speak and understand English or Spanish; all caregivers interviews were done in English, though one caregiver was a bilingual Spanish-speaker. Phase 1 adolescents and caregivers were enrolled independently (i.e., we did not enroll youth and caregivers as dyads).

### Recruitment

A member of the research team approached families during the youth’s virtual or in-person court intake appointment, to ask if they were interested in being contacted about a research study for which they may be eligible to participate. Youth over the age of 18 and caregivers of interested minors were invited to sign a consent-to-contact form or provide verbal consent (as permitted by IRB approved procedures due to COVID-19) allowing the research team to review the youth’s court records for eligibility criteria and approach them for secondary screening and enrollment. When contacting families, the research team member would describe the active phase of the research study and invite interested diverted youth and/or caregivers to complete informed consent; parental consent and child assent were obtained for participants under the age of 18. Phase 1 recruitment began in December 2020 and ended in November 2021. Phase 2 recruitment was conducted from June 2022 to September 2022.

## Phase 1: TECH Prototype Development

### Qualitative Interview Procedures

The primary goal of Phase 1 interviews was to identify the digital intervention needs and preferences of diverted youth who use cannabis, in order to guide initial development of the TECH app. Both caregivers and youth were asked about diverted youth’s technology access and the perceived feasibility and acceptability of digital interventions for diverted youth in general, in the family court context, and for the proposed TECH app. Interview questions covered key dimensions of the Behavior Intervention Technology (BIT) model (Mohr et al., 2014), an integrated conceptual framework that helps digital intervention developers to distill the “what, why, how, and when” of their intervention. Specifically, the guide confirmed “what” behaviors youth wanted help changing and “why” youth needed the proposed app (i.e., clinical and usage aims), “how” the app could help youth change their behavior (i.e., behavior change strategies), and which features would best achieve that objective (i.e., digital elements, design characteristics and workflows). On average, caregiver interviews lasted 24 min (*SD* = 6; range 14–30 min) while interviews with diverted youth lasted 51 min (*SD* = 11; range 40–78 min), reflecting solicitation of end-user feedback about anticipated TECH app features.

Following the BIT model, we specifically asked youth for guidance around five anticipated components of the TECH app we selected a priori to help achieve our theory-driven clinical and app usage aims. We anticipated targeting intrapersonal mechanisms of SU behavior change via features that would enhance user motivation to change SU or directly facilitate a user’s SU behavior change efforts. We sought to target interpersonal mechanisms of SU behavior change via interactive features that allowed supportive, prosocial engagement. Notably, all our anticipated behavior change components were identified as widely used components of digital interventions for young adult cannabis use in a recent meta-analysis (Côté et al., 2024). Finally, to achieve the BIT model’s theory-driven app usage aims—which seek to engage youth with the app enough to expose them to our clinically-oriented components, we anticipated including gamification(see Cugelman, 2013) and personalization components. Following established strategies for mobile app development (e.g., Santesteban-Echarri et al., 2021), diverted youth and caregivers were shown screenshots of several then-publicly available apps for cannabis use reduction or abstinence (e.g., *Quit Cannabis* (Memsta Apps, 2024), *Grounded: Quit Weed Smoking* [Grounded, 2024], *Stop-Cannabis* [Benarous et al., 2016]) to demonstrate how several anticipated TECH app components (i.e., personalization, behavior change, social interaction, gamification, motivation enhancement) might look or function. Diverted youth were also asked to rate the five anticipated components on a scale of perceived importance (1 = *not important* to 10 = *very important*) and select their top 3 must-have components.

All participants completed online surveys before being interviewed; youth completed their online survey, received the court’s TAU, then completed their individual interview. The family court’s TAU for diverted youth who screened positive for cannabis use at intake included a brief computer program called eCHECKUP-To-Go Cannabis (ECTG; San Diego State University Research Foundation, 2009). The program anonymously collects data about the participant’s SU and provides accurate information about peer use (e.g., peer normative feedback) and personalized behavior change recommendations related to their cannabis use. In their interviews, we specifically asked diverted youth to discuss if and how anticipated TECH app features might help them enact any of their personalized ECTG behavior change recommendations, to ensure the resulting app functioned as an adjunct to TAU.

The study’s principal investigator (SAH) conducted all interviews; she is a Non-Hispanic White, cis-gender female researcher with a doctoral degree in clinical psychology. Interactions between the interviewer and participants were limited to recruitment, consent/assent, and scheduling, followed by the one-time interview. Interviews were audio recorded, transcribed verbatim and checked for accuracy by two independent study team members, then de-identified. Formal field notes were not taken and transcripts were not returned to participants for review.

### Quantitative Measures

Participants provided basic demographics to allow for sample characterization. Diverted youth reported their recent behavioral health service usage on the Child and Adolescent Services Assessment (CASA; Burns et al., 1997; Farmer et al., 1994) and characterized their cannabis use on the Marijuana Use Questionnaire (MUQ; Cuttler & Spradlin, 2017; Metrik et al., 2009).

### Analytic Approach

Caregiver and youth interviews were analyzed separately using a directed content analysis approach (Hsieh & Shannon, 2005). Analysis followed an a priori coding scheme derived from the BIT model (Mohr et al., 2014) constructs. The PI and two additional coders began by establishing definitions for each of the BIT model constructs, to form the initial codebook. The coding team independently applied the initial codebook to the same two transcripts, to refine a priori definitions and ensure consensus. Coding team members could also recommend additional, unanticipated codes; the team established definitions for these emergent codes to ensure consensus, resulting in the final version of the codebook. Two coders then independently reviewed all transcripts using the final version of the codebook. The coding team reconvened to discuss any independent codes and achieve consensus.

Quantitative measures were analyzed using percentages or means and standard deviations. For the five anticipated TECH app features that diverted youth rated on importance and selected their top three must-have features, we calculated the average importance rating and reported what percent of diverted youth endorsed a feature as being a must-have.

## Phase 1 Results

Participant demographics are presented in Table 1. Diverted youth and caregivers we interviewed represented 18 distinct households (i.e., four youth and caregivers were from the same household). Importantly, interviewed youth varied in their readiness to make a change in cannabis use in the next year on the MUQ: 5 said they probably or definitely *would try* to make a change, 5 said they probably or definitely *would not try* make a change, and 4 were *unsure*.

**Table 1:** Phase 1 and 2 participant demographics

	Phase 1: Qualitative Interviews				Phase 2: Beta Testing	
	*Caregivers*		*Adolescents*		*Adolescents*	
Characteristic	*n* = 8		*n* = 14		*n = *10	
Age in years (Mean/SD)	43.88	10.78	16.21	1.25	16.50	1.27
Gender Identity						
Female	6	75%	6	43%	6	60%
Male	2	25%	8	57%	4	40%
Sexual Orientation						
Heterosexual	—	—	9	64%	7	70%
Bisexual	—	—	2	14%	2	20%
Gay or lesbian	—	—	2	14%	1	10%
Other	—	—	1	7%	0	0%
Hispanic/Latinx Ethnicity	3	38%	1	7%	2	20%
Race						
White	5	63%	12	86%	8	80%
Black/African-American	1	13%	0	0%	0	0%
Native American/Alaskan Native	1	13%	0	0%	0	0%
Multiracial	0	0%	2	14%	2	20%
Other	1	13%	0	0%	0	0%
Court mandated to TAU	—	—	8	57%	4	40%
Age first used cannabis (Mean/SD; years)	—	—	13.21	2.05	13.00	1.49
Professional services for mental health or SU problems in past 3 months	—	—	9	64%	8	80%
Estimated lifetime cannabis use	—	—				
1-10 times	—	—	0	0%	0	0%
11-50 times	—	—	3	21%	1	10%
51-100 times	—	—	2	14%	2	20%
Over 100 times	—	—	9	64%	7	70%
Married or Partnered	3	38%	—	—	—	—
Associate’s degree (2-year), or higher	4	50%	—	—	—	—
Household Income below $50,000	6	86%	—	—	—	—
Government Assistance (i.e., social security, unemployment, SNAP, Medicare, Medicaid, or welfare)	5	63%	—	—	—	—

Percentages were calculated using available responses and may not reflect the full sample sizes (e.g., a participant selected “prefer not to answer”). All diverted youth and caregivers endorsed cisgender identities (i.e., no participants identified as gender fluid or transgender)

### Qualitative Themes that Emerged during Analysis

Two additional themes were identified during the coding process that were not related to the TECH app: diverted youth’s access to and use of technology and court-related concerns.

#### Technology Access and Use

Both groups indicated that most diverted youth (i.e., self or interviewee’s child) owned their own smartphones (88%) and used them daily for a range of reasons, most often to connect with peers. Youth reported that their rate of engaging with peers via technology was consistent before and during the COVID-19 pandemic, when the interviews occurred. Participants who had not previously used a behavior change app generally agreed that mobile apps had the capacity to support behavior change, particularly for those ready and eager to make a change, as one youth pointed out: *“I feel like apps like this can only work for a certain type of people who have the right mindset going into it.”* About half of the diverted youth had previously tried a behavior change app, either for exercise or mental health (i.e., depression, non-suicidal self-injury, and positivity), and reported having generally positive experiences.

#### Court-Related Concerns

Most caregivers were open to their child receiving the proposed app via the family court and engaging anonymously with an online community of diverted youth. One caregiver recommended that using the app not be mandated or else it *“would be another thing that is like a responsibility that we have to do. I would have to be after her*,* “Did you do this? … Did you go into the app?’”* Several caregivers indicated they would be more comfortable if someone were responsible for monitoring interactive features to protect against possible peer deviance, with one noting *“you don’t know what kind of kid you’re gonna meet [online]… if it was monitored*,* I wouldn’t have a problem.”* Youth were also open to receiving the app through the family court and were comfortable with content moderation, however they felt strongly that the app should not be monitored by court staff: said one teen, *“trying to have somebody from the court system be a part of it*,* I feel like kids would feel that they can’t say things that they would want to if they were there.”* Another suggested court staff presence on the app could hinder motivation to change, explaining *“There’s a lot of kids who do hate their court workers and don’t want to make that change because of that person.”*

### Qualitative Themes that Aligned with the BIT Model

#### Clinical and Usage Aims

Quotes addressing this aspect of the BIT model elucidated diverted youth’s clinical- and technology use-objectives for the TECH app. Most youth agreed that a smartphone app could help them make a change in their cannabis use. When asked how the app could help them enact their personalized behavior change goals, most youth wanted help tracking their use of, and money spent on, cannabis, interacting more with peers who didn’t use cannabis, and making plans for telling friends about their decision to change their use.

Caregivers wanted the app to focus on helping diverted youth reduce their cannabis use, rather than promoting abstinence, by providing educational content and to help teens make incrementally healthier choices. One caregiver said the app should, *“Help you to slow down and then when you’re ready to get to the stage of not smoking it—because they’re going to look at it and say*,* ‘I don’t want to stop smoking’. However*,* you know*,* ‘I could maybe slow down a little*,*’ that’s a good thing*.*”* Caregivers also emphasized the importance of the app serving as an educational tool to support teens in making informed decisions about cannabis use. Another caregiver explained, *“There are some things kids don’t want to tell their parents directly… It’s a hard conversation to have*,* especially if they’re debating whether they want to smoke or not.”*

#### Behavior Change Strategies

Quotes addressing this aspect of the BIT model referenced behavior change strategies that could help diverted youth achieve their clinical and usage aims. Caregivers and youth highlighted the necessity of behavior change strategies that could enhance teens’ motivation to reduce cannabis use. As one teen put it, *“the motivation to do any of this would be the hardest part.”* These strategies included setting small, achievable goals and providing tools for self-monitoring. Caregivers in particular stressed the need for a gradual approach to goal setting. As one caregiver explained, *“Each step has to be easy to start with because if they start off with something that’s too difficult*,* it’s gonna collapse that whole idea of rehab.”* Self-monitoring was another key strategy identified by both groups. Caregivers and youth suggested that features to track cannabis use or related behaviors could provide clarity about consumption patterns and help set realistic goals. One caregiver highlighted the value of accurate tracking, stating, *“Instead of just saying*,* ‘I smoked like two or three times today*,*’ [the app could clarify*,*] ‘No*,* your app said you actually smoked eight times today’… Kind of like ‘Wow.’”* Another pointed to the utility of visual tools to track achievements, noting *“if they see ‘time since when [you __]’ and that keeps adding up*,* it almost gives them a goal to keep it going.”* Diverted youth similarly advocated for motivational features, including those to help teens reflect on life before and after they made a change in cannabis use. Said one youth, *“[The app could] show what you were doing so good before… and then what was wrong*,* and then be like*,* ‘You were doing this’… try to motivate them to get back into those ways.”*

#### Elements

Interviewees described several general elements essential to facilitating behavior change and engagement, including goal setting and tracking, notifications, competitive features like leaderboards and awards, educational resources, and opportunities for peer interaction. Both groups stressed the importance of notifications to remind users to open the app and stay accountable to their goals. One caregiver suggested scheduled reminders, *“every day at a certain time [the app] would shoot them something”* while youth preferred daily notifications *“like*,* ‘keep pushing*,*’ or something like that… Something to motivate you.”*

Caregivers and diverted youth noted that adolescents can be competitive, making gamified features like leaderboards and awards ideal motivators. One caregiver shared, *“My son’s very competitive and like*,* no matter what he does*,* he has to be the absolute best… so something like a leaderboard is actually*,* I think*,* really relatable to specifically teenagers.”* Informative features that deliver educational content, as one caregiver put it, *“like facts about marijuana use… and maybe… statistics of kids that smoked and kids that quit*,*”* were requested by both groups to provide accurate information about the risks of cannabis use, including statistics and the long-term impacts on health and performance.

Finally, and perhaps most importantly, caregivers and diverted youth both expressed a strong preference for social and interactive elements, such as hubs for conversation and progress-tracking tools. One youth wanted interactive features in order to *“connect with other people… or you can vent and get advice from… different types of people.”* Another noted that interactive features could be motivational: *“…the interactions would keep me going*,* like talking to my friends*,* staying in touch*,* seeing progress would help me be motivated.”* Others acknowledged the benefit of fostering a sense of community amongst peers in similar situations. As one caregiver explained, *“I think it’s important to not feel like you’re the only one trying to quit … having support that other people are dealing with the same thing that you’re dealing with.”* Suggested social and interactive elements included, chats, newsfeeds, forums or progress charts, where youth could give and receive peer support around SU behavior change.

#### Feedback About Anticipated Components

 Diverted youth were somewhat divided as to the importance of each of the five theoretically-driven anticipated components, i.e., motivation, behavior change, interaction, gamification, and personalization. Motivation features were overwhelmingly rated as the top must-have; importance ratings averaged 8.8/10 and all but one youth put it in their top three essential elements. As one youth put it, *“it’s not like someone’s gonna download the app just for the awards and stuff. They’re downloading the app to make progress…they’re not downloading it to interact with people because that’s what social media is for*,* so it’s like motivation is the biggest key component.”* In contrast, just half of diverted youth put behavior change (8.3/10), interaction (7.6/10), or gamification (7.1/10) in their top three must-have components. Personalization was the lowest-rated (7.1/10) and least selected must-have component, largely because youth assumed all elements would be programmed to allow for personalization (e.g., they rated behavior change as a must-have and recommended the ability to enter and track one of your own, non-ECTG goals). When commenting on the pros and cons of including a leaderboard, one diverted youth illustrated the interdependence of a user’s preferences, behavior change goals and their must-have features: *“It wouldn’t make me not want to use [the app]*,* I would probably just avoid [the leaderboard]…If you’re like a heavy user and you’re doing well*,* you’re gonna get like mad points. But if I’m just going on*,* trying to casually cut back a little…it’s gonna show me as being worse than my friends even though we’re using [TECH] for different reasons.”*

#### Characteristics and Workflow

Interviewees did not provide many specific recommendations around the look, feel, or timing of the app, other than expressing a strong preference for simplicity and for features that could be easily customized to suit individual preferences. One diverted youth reflected on the value of simplicity, noting *“It’s something that like you can easily use*,* something that keeps you in the loop of things but isn’t too much information. I like the simpler idea of it and how easy it is to go through and talk to your friends.”* Another emphasized their desire for customization when advocating for the opportunity to enter your own behavior change goals, stating, *“…most people would say keep it personalized so you can…track of whatever you’re trying to keep track of or change.”*

### Building the TECH App Prototype

Prototype development began in November 2021 with a series of meetings between the research team and the software developer. We partnered with Mooseworks Software LLC, a US-based single-developer company that uses a library of pre-programmed features to build affordable mobile apps for a range of customers, including clinical researchers (e.g., Becker et al., 2022). Prior to launching the design phase, both parties engaged in institutional contracting and an information technology and security review process, which itself took approximately 6 months. In an initial 2-hour design meeting with Mooseworks, the research team described the TECH app’s clinical aims (i.e., helping youth diverted from JLS-involvement make a change in their cannabis use through interpersonal and intrapersonal mechanisms of behavior change) and summarized the qualitative results from interviewed youth and caregivers related to anticipated features. Key features that were selected for the prototype included (see Supplementary Table 1): a username and login, user settings, a newsfeed, goal setting, daily log and progress charts –both of which captured daily goals achieved, mood ratings, cannabis used, and money spent on cannabis—in-app notifications, a resources page with information about cannabis and 24 crisis lines, a leaderboard, activity-based badges, and a contact us feature to report malfunctions. Over the next month, the developer and the research team engaged in a series of meetings and email conversations to make decisions about characteristics and workflow of included features, referring back to the qualitative data frequently. As an example, in response to feedback from Phase 1 diverted youth that some teens may not be interested in the daily log feature and thus might be driven away by our proposed daily reminders to log their activities, we instead employed daily positive affirmations sourced from publicly available lists (e.g., Harra, 2018) to promote general app engagement rather than engagement with the daily log. Approximately three weeks after the initial meeting, the first iteration of the app prototype interface was available. From there, the research team and developer engaged in several months of iterative design and refinement of specific components.

Beginning in April 2022, the research team conducted several rounds of functionality testing with approximately 10 adult volunteers who were not involved in the design process, to ensure the software was working as intended. Then, the research team engaged in several rounds of a two-week long quality assurance process to test the app using fake user accounts. This process allowed the research team to populate the newsfeed prior to the launch of beta-testing and to confirm that the values users entered into the logs (e.g., user entered a ‘2’ in the daily log for ‘how many times did you use marijuana today?’) was consistent with the data presented back to the user in their progress charts and the metadata available on the administrator platform. In total, the developer “released” approximately 16 versions of the prototype for review and iterative refinement prior to the launch of Phase 2.

## Phase 2: Beta Testing the TECH Prototype

In June 2022, 10 diverted youth were recruited to beta-test the TECH app prototype (see Supplementary Fig. 1 for screenshots) in the context of a non-randomized pilot study. Unlike functionality testing or quality assurance checks, beta-testing engages real users with the app prototype to identify and refine problematic features. The pilot study also allowed the research team to assess preliminary feasibility and acceptability of the prototype and proposed study procedures prior to Phase 3’s randomized clinical trial.

### Procedures

Recruitment and youth eligibility were consistent with Phase 1; caregivers did not participate in Phase 2. To ensure a sufficient user presence for the app’s interactive features, the research team waited to schedule baseline appointments until after at least 4 participants were available to be enrolled. Phase 2 youth attended a baseline appointment in which they completed online baseline surveys, the court’s TAU (i.e., the ECTG program; San Diego State University Research Foundation, 2009), and were then trained to use the prototype TECH app. Youth had access to the TECH app prototype for one month, after which they completed follow-up surveys.

### Measures

*Feasibility* was captured in two ways. First, we calculated the percent of diverted youth who screened as eligible and ultimately participated in beta-testing. Second, we extracted user-level app metadata, including total visits, newsfeed posts, posts liked, and behavioral logging (i.e., goals, mood, cannabis use, and money spent on cannabis), to see if and how youth used the prototype app. Importantly, youth received a brief training from a research team member on how to use the TECH app. During this training, the team member screen shared the TECH app on a study device and demonstrated how to access and use each feature; youth were encouraged to follow along on their own device. In order to account for any directed app use due to this training, we excluded all app use metadata from the app download date.

*Acceptability* of the TECH app was captured two ways. First, diverted youth completed the Mobile Application Rating Scale - User Version (uMARS; Stoyanov et al., 2016) at follow-up. It is a publicly available and widely-used 20-item digital health quality assurance measure developed for and validated with youth. Youth rated the app on a series of item-specific 5-point Likert scales, where higher scores are better; uMARS subscales capture user-reported engagement, functionality, aesthetics, and information. Our target for app quality was average subscale scores of > 3. Second, diverted youth rated their satisfaction with the app on the 11-item Consumer Satisfaction Questionnaire (CSQ; McMahon & Forehand, 2003). Youth rated each question on a series of item-specific, 7-point Likert scales (e.g., 1 = *very dissatisfied* to 7 = *very satisfied*) and an average satisfaction score was calculated. The CSQ ended with an open-response question asking how the TECH app could be improved to help more than it has.

### Analytic Approach

Due to the small sample, quantitative data are only presented in aggregate as percentages or means and standard deviations, to offer insights into the samples and preliminary feasibility and acceptability of the TECH app prototype.

## Phase 2 Results

Participant demographics are presented in Table 1. Most Phase 2 youth were ready to make a change in cannabis use in the upcoming year: seven said they probably or definitely *would try* to make a change, while just three said they probably or definitely *would not try* make a change. Two Phase 2 youth were lost to follow-up; one was placed in a group home two days after baseline and lost access to their phone (and thus, the app prototype), the other could not be reached to schedule their follow up. Importantly, both these youth had post-baseline TECH app metadata, indicating they used the TECH app prototype after their download date.

### Feasibility

#### Participation

Of the 18 diverted youth who screened for eligibility, 11 met criteria; seven were ineligible due to no smartphone access (*n* = 2) or reporting no past-year cannabis use (*n* = 5). Of those 11 eligible youth, 10 enrolled, reflecting a 91% participation rate.

#### Metadata

Means, standard deviations, and ranges are presented in Table 2. All 10 Phase 2 youth independently used the app after their download day; therefore, metadata reflect the full sample (vs. *n* = 8 who completed a follow-up). On average, youth visited the app 19 times, opening the app on 8.1 days. The most used features included posting on the newsfeed and liking a peer’s post, both of which were used by 8 of 10 youth. The daily log feature split data entry across two pages, first asking youth to log their goals and mood, then on the next page youth could enter their cannabis use and/or money spent on cannabis. Seven of 10 youth logged a daily goal or mood, but only 5 logged any cannabis use and only 3 logged money spent on cannabis. Notably, three Phase 2 youth continued to use the app *after* their final study appointment.

**Table 2:** Phase 2 beta testing metadata totals

Diverted Youth		Mandated to TAU?	Try to change cannabis use in the next year?	Days Visited	Visits to App	Posts/ Replies	Likes Given	Days the Youth Logged*			
								Goals	Mood	Cannabis use	Costs
Female, 15		No	Definitely will	3	3	0	0	0	0	0	0
Male, 18		Yes	Definitely will	4	12	2	1	2	2	0	0
Male, 17		Yes	Definitely will	9	16	4	13	0	0	0	0
Female, 17		No	Probably will	6	9	0	0	3	3	1	0
Female, 17		No	Probably will	9	31	4	2	4	4	3	3
Female, 14		No	Probably will	11	31	1	22	8	8	6	1
Male, 18		Yes	Probably will	4	4	2	2	0	0	0	0
Female, 16		No	Probably will not	6	25	2	1	3	3	1	0
Male, 17		No	Probably will not	22	36	3	22	10	10	0	0
Female, 16		No	Probably will not	7	20	7	13	2	2	1	1
	Mean (*SD*)			8.10 (5.22)	18.70 (11.80)	2.50 (2.12)	7.60 (9.06)	3.20 (3.39)	3.20 (3.39)	1.20 (1.93)	0.50 (0.97)
	How many youth engaged?			–	10/10	8/10	8/10	7/10	7/10	5/10	3/10

Metadata reflects the total count of each variable, e.g., total visits to the app or total days on which the youth logged any mood data, during the 30-day beta testing period. We did not include any activity from the app download day, to avoid counting directed app use during the TECH app introduction. Item assessing plans to change cannabis use came from the Marijuana Use Questionnaire (Cuttler & Spradlin, 2017; Metrik et al., 2009); the question asked, “How likely is it that you will try to cut down or stop using marijuana in the next year?” and response options were *definitely will not*, *probably will not*, *unsure*, *probably will*, or *definitely will*. *Youth could log information about goals achieved, mood, cannabis use, and costs up to 7 days retrospectively, such that “days the youth logged” reflects the total days on which a user submitted any data, not how many days of data were obtained for the 30-day beta testing period

### Acceptability

#### App Quality

Average participant ratings on the uMARS, which used a scale of 1 to 5 where higher scores indicate higher quality, indicated the TECH app prototype had very good functionality (*M* = 4.06, *SD* = 0.74) and information (*M* = 4.23, *SD* = 0.67), and acceptable engagement (*M* = 3.13, *SD* = 0.72) and aesthetics (*M* = 3.25, *SD* = 1.15).

*Satisfaction.* On the CSQ, which uses a 1 to 7 scale where higher scores indicated higher satisfaction, satisfaction ratings averaged 4.80 (*SD* = 0.58), indicating participants were somewhat satisfied with the TECH app prototype. Of the six diverted youth who provided free response recommendations to improve the TECH app, feedback largely centered around improving the app’s design (e.g., “make it more colorful, maybe a different layout”) or expanding its functionality (e.g., “adding more customization” and “more color/things to do”).

## Discussion

This study reported on the development and beta-testing of the TECH app, a novel adjunctive digital intervention intended to supplement SU services as-usual received by diverted youth. To our knowledge, TECH is the first SU mobile app to be designed and refined in collaboration with diverted youth and their caregivers, a critical step in digital intervention development that ensures the end product ultimately serves its end users (Mohr et al., 2017). Our research team underwent a three-year user-centered design process, mapping participant feedback directly onto both anticipated, theory-driven components chosen to leverage intrapersonal and interpersonal mechanisms of SU behavior change, as well as additional user-driven components, resulting in a prototype app. This multiphase research led to two principal findings. First, Phase 1 demonstrated clear population-level interest digital adjuncts to help youth diverted away from JLS-involvement make a change in their use of cannabis. Second, the Phase 2 results showed beta-testers found the TECH app feasible and acceptable.

Phase 1 qualitative findings offered a first look into the digital SU intervention needs and preferences of diverted youth, directly informing design decisions for the TECH app prototype. Both caregiver and youth’s preferred features were consistent with must-have features reported in other research on digital intervention development for adolescents (Domin et al., 2022), especially the inclusion of peer interactive features (Ghosh et al., 2024). Perhaps the biggest takeaway from Phase 1 was the variability in youth preferences for anticipated TECH app features and its implications for intervention design. With the exception of motivation enhancing elements, which caregivers also endorsed as an essential feature, youth were split on the importance of all other anticipated features. To some extent, this makes sense—user preferences vary even within subpopulations like diverted youth. Phase 1 youth represented all stages of readiness to make a change in cannabis use, which may partially explain the apparent lack of consensus regarding must-have features. For example, a teen who has no interest in changing their cannabis use would likely not want a logging feature to track their nonexistent behavior change efforts. More importantly, participant feedback underscored the nuances of designing features that could support the broad digital health needs of diverted youth. As was articulated by a study participant, an adolescent cutting back on, rather than quitting, cannabis use may be disillusioned by gamification features that reward total reduction in cannabis used rather than progress towards individual goals. Youth feedback drove the research team to carefully consider the instantiation of each anticipated component—in this case, building gamification features that rewarded behavior change (e.g., achieving goals) and app engagement (e.g., logging goals)—to ensure it would engage most diverted youth who use cannabis.

Phase 2 beta-testing results supported the feasibility and acceptability of the TECH app prototype as a digital tool to address cannabis use among diverted youth. Specifically, the pilot study had a high rate of participation and app engagement, indicating study eligible youth were interested in trying out the app. Metadata showed that all beta-testers visited the app and 80% engaged with one or more features, often on multiple occasions. Visual examination of Table 2 did not provide any obvious explanation for observed variability (i.e., standard deviations) or app use patterns related to gender identity, age, or readiness to change cannabis use, with one exception: youth who visited the app on more beta testing days had higher app use across most features, underscoring the importance of engagement in digital interventions (Perski et al., 2017). Notably, all youth who were mandated to treatment and opted to enroll in the study completed their baseline and follow-up appointments, and their app usage was comparable or superior to non-mandated beta testers, with two exceptions: two mandated youth never used the daily log (versus one non-mandated beta tester) and mandated youth “liked” three fewer posts on average than non-mandated peers. This is especially encouraging, given that at least 25% of downloaded apps are abandoned after their first visit (Ceci, 2023). After having access to the app for one month, participants rated the quality of the app and their satisfaction with it moderately positively. Collectively, beta-testing results indicated the research team could move ahead with the pilot RCT; Phase 3’s pilot RCT (NCT05979272) launched in June 2023 and will test the feasibility, acceptability, and preliminary effectiveness of the beta version of the TECH app in a sample of 60 diverted youth over six months.

### Limitations

This preliminary work must be considered in light of notable limitations. The TECH app was designed in partnership with a single, statewide family court. It was conceptualized as a digital adjunct that could sustain motivation to change among diverted youth who received a brief computerized intervention for cannabis use and help facilitate SU-related behavior change. Diversion programs can vary across jurisdictions and JLS settings, such that generalizability of the TECH app may be limited to settings that offer diverted youth SU services. Our sample was limited to a small number of youth and caregivers recruited entirely from our partner family court; as such, our findings solely reflect the perspectives of constituents and end users in a single-family court and region of the US. Relatedly, our samples had slightly higher prevalences of non-Hispanic White-identifying (i.e., 75% in this study, versus 62% court-wide) and female (i.e., 50% in this study, versus 29% court-wide) diverted youth than previously reported demographics of our partner court’s population (reported in Helseth et al., 2024). This may be an artifact of our study’s focus on cannabis use, the use of which has been shown to differ among various racial and ethnic groups (Substance Abuse and Mental Health Services Administration, 2024), or our small sample sizes (i.e., the difference in our sample’s rate of non-Hispanic White diverted youth and the court overall was by about 3 youth). Due to the emphasis on intervention content and user-centered design, rather than program implementation at the organizational level, we did not collect data from other key constituents like JLS staff or members of the court. Their guidance will be vital to future implementation and scale out efforts, if results are promising.

### Future Directions

Digital interventions offer an accessible and scalable means of delivering clinical services with high-fidelity, particularly in high need, low resource settings that lack a clinically trained in-house workforce, which describes many JLS settings. Even so, the promise of digital approaches hinges on their ability to affect meaningful, real-world changes for youth who use them. Though still in its early stages, this work represents a small but important step forward in the broader effort to design and adapt digital health interventions (Bath et al., 2018) that can serve the unique behavioral health needs of youth in the JLS. A critical next step is to establish the efficacy and effectiveness of the TECH app as an early SU intervention tool for youth diverted from the JLS who use cannabis use; the pilot randomized trial of TECH is currently under way and will offer further insight into the clinical utility of digital interventions for youth in the JLS.

## Supplementary Information

Below is the link to the electronic supplementary material.

Supplementary Material 1

## Data Availability

Aggregate data are available upon request; contact the corresponding author.
