# Supplementary material for: User-centered Design of an Adjunct Smartphone App to Reduce Cannabis Use among Youth Diverted from the Juvenile Legal System
Source: Res Child Adolesc Psychopathol. 2025 Sep 9;53(12):1797–811. doi: 10.1007/s10802-025-01370-6 (PMC12422710; doi:10.1007/s10802-025-01370-6)
Supplement: Supplementary file 1 — Supplementary Material 1 [file 10802_2025_1370_MOESM1_ESM.docx]

**Supplementary Table 1. Mapping Anticipated Components onto Selected Features in the TECH Prototype**

|  | **Anticipated Components** | | | | |
| --- | --- | --- | --- | --- | --- |
| **Selected Feature** | *Motivation* | *Behavior Change* | *Interaction* | *Gamification* | *Personalization* |
| Login & Settings |  |  |  |  | X |
| Newsfeed | X |  | X |  |  |
| Goal Setting | X | X |  |  | X |
| Daily Log & Progress Charts | X | X |  | X | X |
| Badges & Leaderboard | X |  | X | X |  |
| Daily Affirmations & Notifications | X |  | X | X | X |
| Resources & Contact Us | X |  |  |  |  |

**Supplementary Figure 1. Screenshots of TECH App Prototype**

| 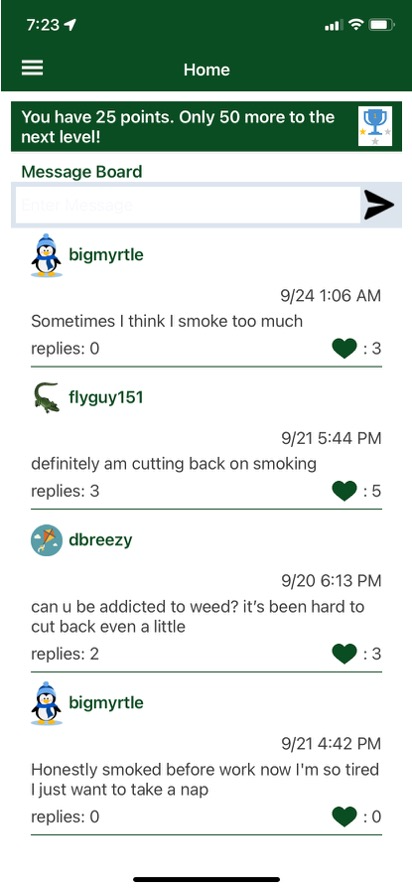 | 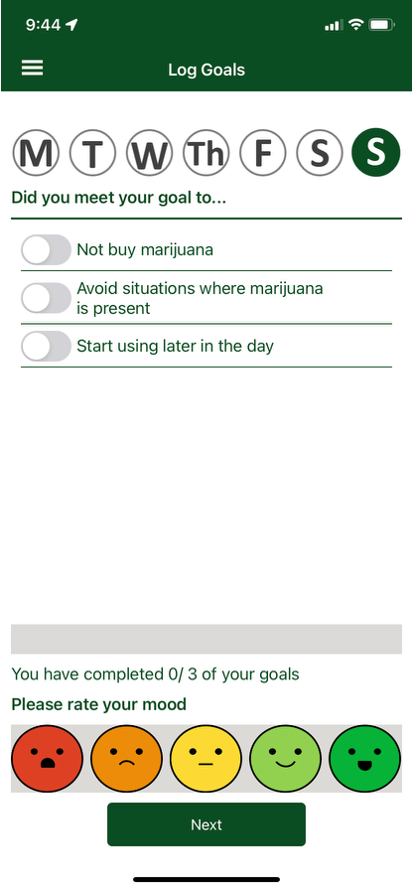 |
| --- | --- |
| 1a. *Homepage and Newsfeed* | 1b. *Daily Goals and Mood Log* |
